# The top 100 most-cited articles on artificial intelligence in breast radiology: a bibliometric analysis

**DOI:** 10.1186/s13244-024-01869-4

**Published:** 2024-12-12

**Authors:** Sneha Singh, Nuala A. Healy

**Affiliations:** 1 Department of Radiology, Royal College of Surgeons in Ireland, Dublin, Ireland; 2 Beaumont Breast Centre, Beaumont Hospital, Dublin, Ireland; 3 Department of Radiology, University of Cambridge, Cambridge, United Kingdom

**Keywords:** Artificial intelligence, Breast, Radiology

## Abstract

**Introduction:**

Artificial intelligence (AI) in radiology is a rapidly evolving field. In breast imaging, AI has already been applied in a real-world setting and multiple studies have been conducted in the area. The aim of this analysis is to identify the most influential publications on the topic of artificial intelligence in breast imaging.

**Methods:**

A retrospective bibliometric analysis was conducted on artificial intelligence in breast radiology using the Web of Science database. The search strategy involved searching for the keywords ‘breast radiology’ or ‘breast imaging’ and the various keywords associated with AI such as ‘deep learning’, ‘machine learning,’ and ‘neural networks’.

**Results:**

From the top 100 list, the number of citations per article ranged from 30 to 346 (average 85). The highest cited article titled ‘Artificial Neural Networks In Mammography—Application To Decision-Making In The Diagnosis Of Breast-Cancer’ was published in *Radiology* in 1993. Eighty-three of the articles were published in the last 10 years. The journal with the greatest number of articles was *Radiology* (*n* = 22). The most common country of origin was the United States (*n* = 51). Commonly occurring topics published were the use of deep learning models for breast cancer detection in mammography or ultrasound, radiomics in breast cancer, and the use of AI for breast cancer risk prediction.

**Conclusion:**

This study provides a comprehensive analysis of the top 100 most-cited papers on the subject of artificial intelligence in breast radiology and discusses the current most influential papers in the field.

**Clinical relevance statement:**

This article provides a concise summary of the top 100 most-cited articles in the field of artificial intelligence in breast radiology. It discusses the most impactful articles and explores the recent trends and topics of research in the field.

**Key Points:**

- Multiple studies have been conducted on AI in breast radiology.
- The most-cited article was published in the journal *Radiology* in 1993.
- This study highlights influential articles and topics on AI in breast radiology.

**Graphical Abstract:**

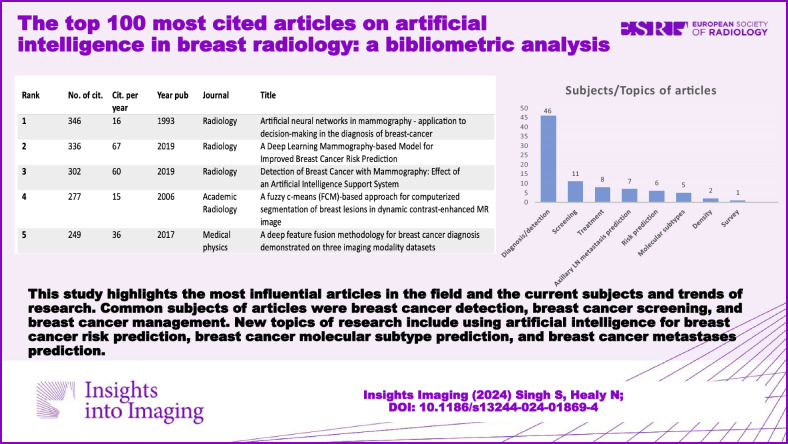

## Introduction

In broad terms, artificial intelligence (AI) is described as the ability of a machine or computer to carry out cognitive tasks and display intelligent behaviour that mirrors that of humans [1, 2]. Back in the 1950s, Alan Turing introduced the concept of the ‘imitation game’ and the Turing test is now used to test a machine’s ability to exhibit intelligent behaviour [3]. AI is not a novel concept and the term ‘artificial intelligence’ was initially suggested by John McCarthy to the computer science community in 1956 at Dartmouth College, USA [4]. In more recent years, the field of AI has been rapidly expanding and is being applied in real-world settings in a variety of scenarios, including medicine.

The umbrella term of AI encompasses many facets with considerable evolution from the initial descriptions in the 1950s. The modern era of artificial intelligence is based on the technologies of deep learning and machine learning. Machine learning uses statistical algorithms and data to allow AI to parallel the way humans learn. Neural networks are programs or models inspired by the neural networks in the human brain that make decisions in a manner similar to humans [5]. Deep learning is a more sophisticated version of machine learning using multi-layered neural networks that allows a computer to train itself to recognise patterns [1, 5].

The use of AI in healthcare is a dynamic and rapidly progressing field of research and innovation. In the US in 2023, 21% of the total venture capital healthcare funds were invested in AI healthcare companies [6]. In the field of radiology, multiple studies have revealed promising results for the use of AI in medical imaging, especially in oncology imaging [7]. The use of artificial intelligence in breast radiology is a topic of growing interest and has already been applied in a real-world setting. A Swedish prospective study of 55,581 women who underwent population-based screening for breast cancer showed that replacing one radiologist with AI was non-inferior for cancer detection compared with double reading by two radiologists [8].

A bibliometric analysis is a statistical evaluation of publications to assess the impact of them. Bibliometrics can be useful in identifying trends in a specific field of research and identifying gaps where further research is required [9]. To the best of our knowledge, there have been no bibliometric analyses conducted focusing on the topic of AI in breast radiology. Hence, the aim of this bibliometric analysis is to identify the top 100 most-cited articles on artificial intelligence in breast radiology across all scientific journals. We present the most influential publications on this topic to date and the current research trends and subjects in this field.

## Methods

The bibliometric analysis was conducted in July 2024. Ethical approval was not required for this study as it is a retrospective bibliometric analysis, not involving patients. Articles were identified using the Web of Science (WoS, Clarivate Analytics, Philadelphia, USA) citation indexing service. Journal impact factors were obtained from the Clarivate ‘Journal Citation Reports’ 2023 database.

The search strategy involved searching for articles that contained the words ‘breast radiology’ or ‘breast imaging’ and the words ‘artificial intelligence’ and its many synonyms. The following search strategy was used to identify journal articles: ‘breast radiology’ OR ’breast imaging’ AND ‘artificial intelligence’ OR “deep learning” OR “computer aided diagnosis” OR “machine intelligence” OR “machine learning” OR “natural language processing” OR “support vector machine” OR “naïve Bayes” OR “Bayesian learning” OR “artificial neural network” OR “neural network” OR “convolutional neural network” OR “random forest” OR “k-nearest neighbour” OR “decision tree learning” OR “data mining” OR “fuzzy” OR “fuzzy C-means” OR “computational intelligence” OR “computer reasoning”.

In total, the search returned 1398 articles and these articles were ranked in order of citation count from highest to lowest. One reviewer (S.S.) analyzed each article and collected data relating to the year of publication, journal of publication, number of citations, impact factor of journal, keywords of article, and subject of article.

Articles were included if their title, abstract or keywords included the search terms of interest. The articles were ranked in order of citation count, from highest to lowest. The top 100 articles were reviewed. The following information was collected from each article: title, year of publication, journal published in, author institution, country, article type, keywords and journal impact factor. Articles were divided into research or review articles.

The full text of each article was analyzed in order to identify the subject of the article. Keywords were extracted from each article. Journal impact factors were obtained from the Clarivate ‘Journal Citation Reports’ 2023 database and the number of citations per year was also calculated by dividing the total citations by the number of years since publication.

## Results

This study identified the top 100 most-cited articles in AI in breast imaging [10–109]. The top 20 most-cited articles are illustrated in Table 1. The article with the highest number of total citations was ‘Artificial Neural Networks in Mammography—Application to Decision-Making in The Diagnosis of Breast-Cancer’ published in the journal *Radiology* in 1993 with 346 citations [10]. This was followed by the article ‘A Deep Learning Mammography-based Model for Improved Breast Cancer Risk Prediction’ published in *Radiology* in 2019 with 336 citations [11]. This article has had 67 citations per year which makes it the AI in breast imaging article with the highest number of citations per year. In the top 100 list of most-cited articles, the average number of total citations was 85 (range 30–346), and the average number of citations per year was 17 (range 2–67).

**Table 1:** Characteristics of the top 20 most-cited articles on artificial intelligence in breast radiology

Rank	No. of citations	Citations per year	Year of publication	Subject	Journal	Title
1	346	16	1993	Breast cancer diagnosis/detection on MG	*Radiology*	Artificial neural networks in mammography—application to decision-making in the diagnosis of breast-cancer
2	336	67	2019	Breast cancer risk prediction	*Radiology*	A deep learning mammography-based model for improved breast cancer risk prediction
3	302	60	2019	Breast cancer diagnosis/detection on MG	*Radiology*	Detection of breast cancer with mammography: effect of an artificial intelligence support system
4	277	15	2006	Breast cancer diagnosis/detection on MR	*Academic Radiology*	A fuzzy c-means (FCM)-based approach for computerized segmentation of breast lesions in dynamic contrast-enhanced MR image
5	249	36	2017	Breast cancer diagnosis/detection in all modalities	*Medical Physics*	A deep feature fusion methodology for breast cancer diagnosis demonstrated on three imaging modality datasets
6	230	32	2017	Breast cancer diagnosis/detection on MG	*Investigative Radiology*	Deep learning in mammography diagnostic accuracy of a multipurpose image analysis software in the detection of breast cancer
7	207	10	2003	Breast cancer diagnosis/detection on US	*Radiology*	Breast lesions on sonograms: computer-aided diagnosis with nearly setting-independent features and artificial neural networks
8	187	12	2008	Breast cancer diagnosis/detection on MR	*Academic Radiology*	Quantitative analysis of lesion morphology and texture features for diagnostic prediction in breast MRI
9	187	47	2020	Breast cancer metastasis prediction	*Radiology*	Lymph node metastasis prediction from primary breast cancer US images using deep learning
10	177	6	1995	Breast cancer diagnosis/detection on MG	*Radiology*	Breast-cancer—prediction with artificial neural-network-based on bi-rads standardized lexicon
11	174	35	2019	Breast cancer treatment	*Investigative Radiology*	Impact of machine learning with multiparametric magnetic resonance imaging of the breast for early prediction of response to neoadjuvant chemotherapy and survival outcomes in breast cancer patients
12	158	32	2019	Review article	*Radiology*	Artificial intelligence for mammography and digital breast tomosynthesis: current concepts and future perspectives
13	155	31	2019	Breast density	*Radiology*	Mammographic breast density assessment using deep learning: clinical implementation
14	153	31	2019	Radiomics	*Radiology*	Radiomic versus convolutional neural networks analysis for classification of contrast-enhancing lesions at multiparametric breast MRI
15	147	29	2019	Review	*Clinical Radiology*	Artificial intelligence in breast imaging
16	147	37	2020	Radiomics	*Breast*	Overview of radiomics in breast cancer diagnosis and prognostication
17	130	26	2019	Radiomics	*European Radiology*	Radiomic nomogram for prediction of axillary lymph node metastasis in breast cancer
18	127	9	2010	Breast cancer risk prediction	*Radiographics*	Informatics in radiology comparison of logistic regression and artificial neural network models in breast cancer risk estimation
19	118	24	2019	Breast cancer diagnosis/detection on US	*Japanese Journal of Radiology*	Distinction between benign and malignant breast masses at breast ultrasound using deep learning method with convolutional neural network
20	111	22	2019	Screening	*European Radiology*	Can we reduce the workload of mammographic screening by automatic identification of normal exams with artificial intelligence? A feasibility study

*MG* mammogram, *MR* magnetic resonance, *US* ultrasound

A total of 29 journals contributed to the top 100 most-cited list. The journal with the highest number of articles in the top 100 list was *Radiology* with 22 publications. This was followed by *European Radiology* with 20 publications and *Academic Radiology* with 11 publications.

The journal with the highest impact factor was *Radiology* with an impact factor of 12.1 as per the Clarivate ‘Journal Citation Reports’ 2023.

A total of 83 of the 100 articles were published in the last decade (2014 and onwards). Only 17 were published before 2014, with the earliest, and also most cited, article published in 1993 in *Radiology* titled ‘Artificial Neural Networks in Mammography—Application to Decision-Making in The Diagnosis of Breast-Cancer’ [10]. The most recently published article on the top 100 list was published in 2023 in *Radiology*, titled ‘Automation Bias in Mammography: The Impact of Artificial Intelligence BI-RADS Suggestions on Reader Performance’ with 50 citations [72].

The articles in the top 100 list were produced by 63 different institutions. The greatest number of articles were contributed by the University of Chicago (*n* = 8), followed by Harvard University, Huazhong University and Radboud University Nijmegen, which all contributed five articles each. The country that provided the highest number of publications was the USA (*n* = 51) followed by China (*n* = 17) and the Netherlands (*n* = 6).

**Fig. 1:**
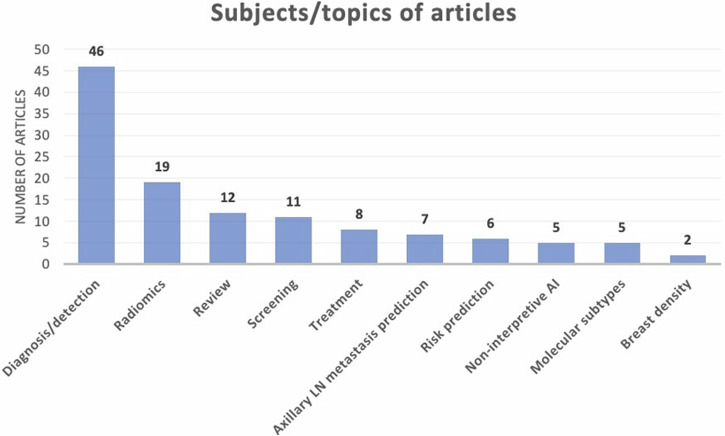
Subjects/topics of the top 100 most-cited articles on artificial intelligence in breast radiology. Shown are the most common subjects covered by the articles in the top 100 list on the *x*-axis and the number of articles on the *y*-axis. Breast cancer diagnosis/detection was the most common subject discussed (*n* = 46), followed by radiomics (*n* = 19), review articles (*n* = 12) and breast cancer screening (*n* = 11). Note some articles fall into more than one category

**Fig. 2:**
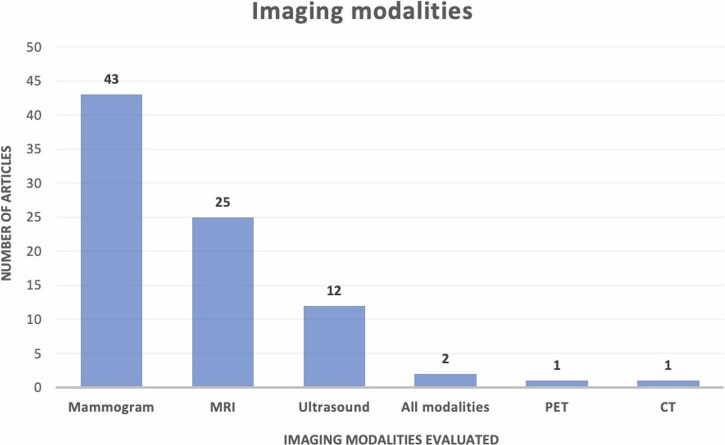
Imaging modalities evaluated in the top 100 most-cited articles on artificial intelligence in breast radiology. Shown are the imaging modalities evaluated in the articles in the top 100 list on the *x*-axis and the number of articles on the *y*-axis. Mammography was the most common imaging modality explored (*n* = 43) followed by MRI (*n* = 25) and ultrasound (*n* = 12). All modalities include US, MRI and mammography. Note 16 articles were excluded from this figure as they did not focus on an imaging modality (review articles, survey articles, etc.)

Of the 100 most-cited articles (electronic supplementary material), twelve were review articles. Among the original articles, the most common subject was the use of AI in breast cancer detection and diagnosis (*n* = 46) (Fig. 1). This can be further divided into breast cancer diagnosis using mammography (*n* = 20), MRI (*n* = 18), ultrasound (*n* = 8) and using all three modalities (*n* = 2) (Fig. 2). The article titled ‘Detection of Breast Cancer with Mammography: Effect of an Artificial Intelligence Support System’ published in *Radiology* in 2019 was the highest cited article in this subject with 302 citations [12]. The other most common subjects included AI in breast cancer screening (*n* = 11), the use of AI in axillary lymph node metastasis prediction (*n* = 7) and the use of AI in breast cancer molecular subtype prediction (*n* = 5) (see Fig. 1).

As AI is an evolving field, with interest in the subject in relation to breast imaging exploding in the past few years, we also opted to evaluate the most-cited articles over the past 5 years to potentially incorporate the more novel AI techniques and some of the larger trials on the topic (see Table 2). The article with the greatest total number of citations and citations per year was ‘A Deep Learning Mammography-based Model for Improved Breast Cancer Risk Prediction’ published in *Radiology* in 2019 with 336 citations and 67 citations per year [11].

**Table 2:** Characteristics of the top 20 most-cited articles on artificial intelligence in breast radiology in the last 5 years

Rank	No. of citations	Citations per year	Year of publication	Subject	Journal	Article
1	336	67	2019	Breast cancer risk prediction	*Radiology*	A deep learning mammography-based model for improved breast cancer risk prediction
2	302	60	2019	Breast cancer diagnosis/detection on MG	*Radiology*	Detection of breast cancer with mammography: effect of an artificial intelligence support system
3	187	47	2020	Breast cancer metastasis prediction	*Radiology*	Lymph node metastasis prediction from primary breast cancer US images using deep learning
4	174	35	2019	Breast cancer treatment	*Investigative Radiology*	Impact of machine learning with multiparametric magnetic resonance imaging of the breast for early prediction of response to neoadjuvant chemotherapy and survival outcomes in breast cancer patients
5	158	32	2019	Review article	*Radiology*	Artificial intelligence for mammography and digital breast tomosynthesis: current concepts and future perspectives
6	155	31	2019	Breast density	*Radiology*	Mammographic breast density assessment using deep learning: clinical implementation
7	153	31	2019	Radiomics, Breast cancer diagnosis/detection on MR	*Radiology*	Radiomic versus convolutional neural networks analysis for classification of contrast-enhancing lesions at multiparametric breast MRI
8	147	29	2019	Review article	*Clinical Radiology*	Artificial intelligence in breast imaging
9	147	37	2020	Radiomics, Breast cancer diagnosis/detection all modalities	*Breast*	Overview of radiomics in breast cancer diagnosis and prognostication
10	130	26	2019	Radiomics, Breast cancer metastasis prediction	*European Radiology*	Radiomic nomogram for prediction of axillary lymph node metastasis in breast cancer
11	118	24	2019	Breast cancer diagnosis/detection on US	*Japanese Journal of Radiology*	Distinction between benign and malignant breast masses at breast ultrasound using deep learning method with convolutional neural network
12	111	22	2019	Screening	*European Radiology*	Can we reduce the workload of mammographic screening by automatic identification of normal exams with artificial intelligence? A feasibility study
13	108	22	2019	Screening	*Radiology*	A deep learning model to triage screening mammograms: a simulation study
14	102	20	2019	Breast cancer diagnosis/detection on MG	*Radiology Artificial Intelligence*	Improving accuracy and efficiency with concurrent use of artificial intelligence for digital breast tomosynthesis
15	92	23	2020	Breast cancer diagnosis/detection on MR	*Scientific Reports*	A deep learning methodology for improved breast cancer diagnosis using multiparametric MRI
16	90	18	2019	Breast cancer diagnosis/detection on MG	*Radiology*	Predicting breast cancer by applying deep learning to linked health records and mammograms
17	88	22	2020	Review article	*British Journal of Radiology*	CAD and AI for breast cancer-recent development and challenges
18	86	22	2020	Breast cancer diagnosis/detection on MR	*Investigative Radiology*	Artificial intelligence-based classification of breast lesions imaged with a multiparametric breast MRI protocol with ultrafast DCE-MRI, T2, and DWI
19	86	22	2020	Breast cancer risk prediction	*Radiology*	Comparison of a deep learning risk score and standard mammographic density score for breast cancer risk prediction
20	82	16	2019	Breast cancer diagnosis/detection on MR	*Journal of MRI*	Weakly supervised 3D deep learning for breast cancer classification and localization of the lesions in MR images

*MG* mammogram, *MR* magnetic resonance, *US* ultrasound

## Discussion

In this paper, we report the findings of a bibliometric analysis that we conducted on the topic of artificial intelligence in breast imaging. This bibliometric analysis provides insight into the use of AI in breast radiology. It showcases the many institutions, journals, subject areas and countries that have contributed most to this field and provides an insight into recent developments in the field. This bibliography showcases the many advances in AI in breast radiology in the last decade not only in the subject of breast cancer detection/screening but also in breast cancer risk prediction, prognosis and treatment. The most-cited article was ‘Artificial Neural Networks in Mammography—Application to Decision-Making in The Diagnosis of Breast-Cancer’, published, like the majority of the articles in the Top 100, in *Radiology* journal [10].

The comparison of an AI system’s performance in interpretation of breast imaging versus that of a radiologist’s remains a topical and highly researched subject [12, 15, 28, 34]. A highly cited study published in 2017 in *Investigative Radiology* found that AI tools demonstrated similar diagnostic accuracy in detection of breast cancer on mammography to that of radiologists [15]. This was supported by a recent study published in 2023 by Chen et al comparing the performance of an AI system with 552 human readers that found no difference in performance when interpreting Personal Performance in Mammographic Screening (PERFORMS) test sets, enriched sets that radiologists use to monitor their performance [110].

More recently, work has been published investigating the ability of AI to support the reading of mammograms by a radiologist [12, 47, 111] The improvement of a breast radiologist’s diagnostic performance was demonstrated by one of the most highly cited articles ‘Detection of Breast Cancer with Mammography: Effect of an Artificial Intelligence Support System’ [12]. The findings of this study showed a higher area under the receiver operating characteristic curve, improved sensitivity and specificity without additional reading time when AI support for radiologists was utilized when reading mammograms.

Many studies have found that applying a deep learning model to breast cancer screening can lead to a reduction in workload with improved specificity [29, 30, 52]. One particular study published in 2021 demonstrated a 70% reduction in screening workload in mammography and digital breast tomosynthesis (DBT) after employing an AI system without reducing the sensitivity being the first of its kind at the time to show this effect in DBT as well as digital mammography [52]. However, this study also highlighted the risk of missing cancers when using AI tools for screening raising questions over the ethical challenges of using AI in breast screening. A study by Rodriguez-Ruiz et al found that using a low cancer detection threshold of an AI system led to a workload reduction of 17% while only excluding 1% of true-positive scans, while a higher threshold leading to 7% of positive scans being excluded [29]. Hence, it can be said that although AI serves as a useful cognitive companion for breast radiologists, it does not demonstrate a superior performance to a radiologist in all scenarios and may have some disadvantages.

Computer-aided diagnosis (CAD) appeared frequently in the results of this search, as a subject of the articles themselves and as a keyword. The term CAD first appeared during the second era of AI in the 1980s and 1990s and was widely used in screening mammography. By 2010, it was estimated that more than 74% of mammograms in the United States were read with CAD assistance. Unfortunately, its outcomes in breast radiology were disappointing as it led to reduced radiologist accuracy and an increase in the time taken to read a study due to the many false positives it produced [112]. Deep learning, part of the current era of AI, is a more advanced technology that greatly outperforms CAD and is the focus of this bibliography.

Radiomics accounted for 19 papers in the top 100 list. Radiomics involves extracting numerous quantitative features from imaging and converting these digital medical images into minable, high-dimensional data. Analysis of this data can offer insightful information that can enhance our understanding of disease and offer clinical decision support. The highest cited radiomics article with 153 citations titled ‘Radiomic versus Convolutional Neural Networks Analysis for Classification of Contrast-enhancing Lesions at Multiparametric Breast MRI’ identified that a convolutional neural network was superior to radiomic analysis for classifying enhancing lesions as benign or malignant on multiparametric breast MRI in a study group of 447 patients [23]. Five of the 19 articles explored applying radiomics to breast cancer diagnosis/detection [23, 25, 64, 70, 81]. Another five looked at radiomics and breast cancer treatment, mainly focusing on predicting response to neoadjuvant chemotherapy [57, 65, 76, 88, 103]. Four articles applied radiomics to axillary lymph node metastasis prediction [26, 87, 98, 102] while another four articles applied radiomics to breast cancer molecular subtype prediction [43, 49, 67, 75]. There was one scoping review article titled ‘Recent Radiomics Advancements in Breast Cancer: Lessons and Pitfalls for the Next Future’ [106].

Using deep learning and radiomics for the prediction of breast cancer molecular subtypes from imaging features was a subject covered by five articles in the top 100 list [43, 49, 75, 84, 101]. Extracting features from breast MRI for this purpose appears to be a highly researched area however, the other imaging modalities have also been explored more recently. A study by Wenjuan et al published in 2019 involved extracting radiomic features from mammograms and using deep learning algorithms to assess if these features were associated with particular molecular subtypes of breast cancer, such as HER2 positive or triple negative. They found that triple negative cancers may be rounder, with a regular outline compared to other subtypes and that luminal lesions may be smoother than HER2 positive lesions [43].

Breast cancer risk prediction models was the topic of a number of recent articles in this list [11, 27, 39, 73, 74]. A study conducted by MIT published in 2019 compared three risk prediction models; a conventional clinical risk factor-based model that used risk factors alone, a deep learning model that used mammograms alone and a third model that combined both. The hybrid model substantially improved risk discrimination compared with an already established conventional breast cancer risk model [11]. This article is the second most-cited article overall, and the most cited over the past 5 years.

A total of twelve review articles were included in the top 100 list. One of these was a systematic review looking at AI in breast MRI [97], and the remaining were scoping reviews [21, 24, 36, 37, 45, 53, 56, 63, 80, 96, 106]. Five of the scoping reviews were focused articles on the topics of radiomics in breast cancer [106], AI and mammography [21], AI and breast cancer screening [45], AI in breast ultrasound [63] and AI in breast MRI [80]. The remaining review articles referenced above discussed AI and breast imaging in general.

There were also a number of articles looking at non-interpretive AI in the context of breast imaging. One paper with 108 citations published in *Radiology* used an AI software to triage screening mammograms [30]. Another paper used a neural network to see the effect of patient history data to predict breast cancer [74] while three papers assessed the ability of AI to extract information from radiology reports [10, 77, 78].

From completing this bibliometric analysis, we have identified the wide variety of research that is being conducted on the topic of AI in breast radiology. We have identified a limited number of studies looking at patient perceptions on the use of AI in breast radiology. In the top 100 list, there is only one article looking at women’s preferences on using AI for breast screening [92], and we did not identify any similar articles looking at a symptomatic breast cohort. As well as this, there were a small number of studies in the top 100 list that applied AI to breast cancer treatment [20, 32, 90] (predicting response to chemotherapy and predicting pathological upgrade), so this could be a subject that could be further explored. Furthermore, we did not see any paper looking at applying AI for breast cancer follow-up post-treatment. As AI is a rapidly evolving field with multiple applications in breast imaging, there are a number of topics that do not factor in the topic 100, primarily as they are so novel, and still acquiring citations. These topics include generative AI, large language models, explainable AI and many others.

There are a number of limitations of this study. First, while we endeavoured to be extensive with the search terms used, there may be other important studies that could have been excluded. Our search strategy included broad and specific search terms to attempt to reduce the chance of missing significant articles. Hence, this analysis should not be considered as a complete list of the top 100 publications in the field of AI in breast radiology. During the analysis, we noted that the older articles generated considerably more citations. For this reason, we calculated the number of citations per year to avoid this bias. We used impact factors from 2023 for this analysis, and our study does not account for the fact that these impact factors may have changed since then. The list does not include some of the recently published prospective AI screening trials such as the study titled ‘Artificial intelligence for breast cancer detection in screening mammography in Sweden: a prospective, population-based, paired-reader, non-inferiority study’ by Dembrower et al published in 2023 [8] as not enough time has elapsed post-publication to generate significant citations. Generative AI is an exciting new area with many potential uses in breast radiology. As it is a relatively novel topic, published articles have not made it to the highly cited articles list. Finally, the study did not factor in the chance of potential bias due to the author’s self-citation.

In conclusion, this study provides a detailed analysis of the top 100 articles in the field of artificial intelligence and breast radiology. It gives an insight into the journals, institutions, and subjects that have contributed to the field and discusses the new advancements and popular topics of research in the field in recent times.

## Supplementary information

ELECTRONIC SUPPLEMENTARY MATERIAL

## Data Availability

The material used for this analysis can be found on the Web of Science (WoS, Clarivate Analytics, Philadelphia, USA) citation indexing service and the Clarivate ‘Journal Citation Reports’ 2023 database.

## Abbreviations

AI: Artificial intelligence
CAD: Computer-aided diagnosis
DBT: Digital breast tomosynthesis
