# Supplementary material for: The top 100 most-cited articles on artificial intelligence in breast radiology: a bibliometric analysis
Source: Insights Imaging. 2024 Dec 12;15:297. doi: 10.1186/s13244-024-01869-4 (PMC11638451; doi:10.1186/s13244-024-01869-4)
Supplement: Supplementary file 1 — ELECTRONIC SUPPLEMENTARY MATERIAL [file 13244_2024_1869_MOESM1_ESM.pdf]

The top 100 most cited articles on artificial intelligence in breast radiology: a bibliometric analysis
ELECTRONIC SUPPLEMENTARY MATERIAL

Insights Imaging (2024) Singh S, Healy N.

Rank	No. of cit.	Cit. per year	Year pub	Subject	Journal	Title
**1**	346	16	1993	Breast cancer diagnosis/detection on MG	Radiology	Artificial neural networks in mammography - application to decision-making in the diagnosis of breast-cancer
**2**	336	67	2019	Breast cancer risk prediction	Radiology	A Deep Learning Mammography-based Model for Improved Breast Cancer Risk Prediction
**3**	302	60	2019	Breast cancer diagnosis/detection on MG	Radiology	Detection of Breast Cancer with Mammography: Effect of an Artificial Intelligence Support System
**4**	277	15	2006	Breast cancer diagnosis/detection on MR	Academic Radiology	A fuzzy c-means (FCM)-based approach for computerized segmentation of breast lesions in dynamic contrast-enhanced MR image
**5**	249	36	2017	Breast cancer diagnosis/detection all modalities	Medical physics	A deep feature fusion methodology for breast cancer diagnosis demonstrated on three imaging modality datasets
**6**	230	32	2017	Breast cancer diagnosis/detection on MG	Investigative radiology	Deep Learning in Mammography Diagnostic Accuracy of a Multipurpose Image Analysis Software in the Detection of Breast Cancer
**7**	207	10	2003	Breast cancer diagnosis/detection on US	Radiology	Breast lesions on sonograms: Computer-aided diagnosis with nearly setting-independent features and artificial neural networks
**8**	187	12	2008	Breast cancer diagnosis/detection on MR	Academic Radiology	Quantitative Analysis of Lesion Morphology and Texture Features for Diagnostic Prediction in Breast MRI
**9**	187	47	2020	Breast cancer metastasis prediction	Radiology	Lymph Node Metastasis Prediction from Primary Breast Cancer US Images Using Deep Learning
**10**	177	6	1995	Breast cancer diagnosis/detection on MG	Radiology	Breast-cancer - prediction with artificial neural-network-based on bi-rads standardized lexicon
**11**	174	35	2019	Breast cancer treatment	Investigative Radiology	Impact of Machine Learning With Multiparametric Magnetic Resonance Imaging of the Breast for Early Prediction of Response to Neoadjuvant Chemotherapy and Survival Outcomes in Breast Cancer Patients
**12**	158	32	2019	Review article	Radiology	Artificial Intelligence for Mammography and Digital Breast Tomosynthesis: Current Concepts and Future Perspectives
**13**	155	31	2019	Breast density	Radiology	Mammographic Breast Density Assessment Using Deep Learning: Clinical Implementation
**14**	153	31	2019	Radiomics, Breast cancer diagnosis/detection on MR	Radiology	Radiomic versus Convolutional Neural Networks Analysis for Classification of Contrast-enhancing Lesions at Multiparametric Breast MRI
**15**	147	29	2019	Review article	Clinical Radiology	Artificial intelligence in breast imaging
**16**	147	37	2020	Radiomics, Breast cancer diagnosis/detection all modalities	Breast	Overview of radiomics in breast cancer diagnosis and prognostication
**17**	130	26	2019	Radiomics, Breast cancer metastasis prediction	European Radiology	Radiomic nomogram for prediction of axillary lymph node metastasis in breast cancer
**18**	127	9	2010	Breast cancer risk prediction	Radiographics	Informatics in Radiology Comparison of Logistic Regression and Artificial Neural Network Models in Breast Cancer Risk Estimation
**19**	118	24	2019	Breast cancer diagnosis/detection on US	Japanese Journal of Radiology	Distinction between benign and malignant breast masses at breast ultrasound using deep learning method with convolutional neural network
**20**	111	22	2019	Screening	European Radiology	Can we reduce the workload of mammographic screening by automatic identification of normal exams with artificial intelligence? A feasibility study
**21**	108	22	2019	Screening	Radiology	A Deep Learning Model to Triage Screening Mammograms: A Simulation Study

**22**	102	20	2019	Breast cancer diagnosis/detection on MG	Radiology: Artificial Intelligence	Improving Accuracy and Efficiency with Concurrent Use of Artificial Intelligence for Digital Breast Tomosynthesis
**23**	100	17	2018	Breast cancer treatment	Radiology	High-Risk Breast Lesions: A Machine Learning Model to Predict Pathologic Upgrade and Reduce Unnecessary Surgical Excision
**24**	100	17	2018	Breast cancer diagnosis/detection on US	British Journal of Radiology	Classification of breast cancer in ultrasound imaging using a generic deep learning analysis software: a pilot study
**25**	92	23	2020	Breast cancer diagnosis/detection on MR	Scientific Reports	A deep learning methodology for improved breast cancer diagnosis using multiparametric MRI
**26**	90	18	2019	Breast cancer diagnosis/detection on MG	Radiology	Predicting Breast Cancer by Applying Deep Learning to Linked Health Records and Mammograms
**27**	88	22	2020	Review article	British Journal of Radiology	CAD and AI for breast cancer-recent development and challenges
**28**	87	15	2018	Review article	British Journal of Radiology	Deep learning beyond cats and dogs: recent advances in diagnosing breast cancer with deep neural networks
**29**	86	22	2020	Breast cancer diagnosis/detection on MR	Investigative radiology	Artificial Intelligence-Based Classification of Breast Lesions Imaged With a Multiparametric Breast MRI Protocol With Ultrafast DCE-MRI, T2, and DWI
**30**	86	22	2020	Breast cancer risk prediction	Radiology	Comparison of a Deep Learning Risk Score and Standard Mammographic Density Score for Breast Cancer Risk Prediction
**31**	85	8	2013	Breast cancer diagnosis/detection on MG	International journal of computer assisted radiology & surgery	An evaluation of image descriptors combined with clinical data for breast cancer diagnosis
**32**	82	16	2019	Breast cancer diagnosis/detection on MR	Journal of Magnetic Resonance Imaging	Weakly supervised 3D deep learning for breast cancer classification and localization of the lesions in MR images
**33**	79	16	2019	Breast cancer diagnosis/detection on US	European Radiology	Automatic classification of ultrasound breast lesions using a deep convolutional neural network mimicking human decision-making
**34**	78	16	2019	Radiomics, molecular subtypes	Academic Radiology	Breast Cancer Molecular Subtype Prediction by Mammographic Radiomic Features
**35**	73	3	2003	Breast cancer diagnosis/detection on US	Academic Radiology	Support vector machines for diagnosis of breast tumors on US images
**36**	73	18	2020	Review article	Radiology	Novel Approaches to Screening for Breast Cancer
**37**	73	14	2019	Breast cancer diagnosis/detection on MR	Diagnostic & Interventional radiology	Detection and characterization of MRI breast lesions using deep learning
**38**	72	14	2019	Breast cancer diagnosis/detection on US	Korean Journal of Radiology	Effect of a Deep Learning Framework-Based Computer-Aided Diagnosis System on the Diagnostic Performance of Radiologists in Differentiating between Malignant and Benign Masses on Breast Ultrasonography
**39**	71	3	1997	Breast cancer diagnosis/detection on MG	Artificial Intelligence in Medicine	Fuzzy logic in computer-aided breast cancer diagnosis: analysis of lobulation
**40**	70	12	2018	Radiomics, molecular subtypes	Clinical Radiology	Breast cancer Ki67 expression prediction by DCE-MRI radiomics features
**41**	70	5	2009	Breast cancer diagnosis/detection on MR	Academic Radiology	Prediction of Malignant Breast Lesions from MRI Features: A Comparison of Artificial Neural Network and Logistic Regression Techniques

**42**	70	12	2018	Radiomics, Breast cancer diagnosis/detection on MG	Radiology	Radiomics Based on Adapted Diffusion Kurtosis Imaging Helps to Clarify Most Mammographic Findings Suspicious for Cancer
**43**	68	23	2021	Screening	Radiology	AI-based Strategies to Reduce Workload in Breast Cancer Screening with Mammography and Tomosynthesis: A Retrospective Evaluation
**44**	68	14	2019	Review article	American Journal of Roentgenology	Artificial Intelligence in Breast Imaging: Potentials and Limitations
**45**	68	17	2021	Breast cancer diagnosis/detection on MR	European Radiology	Prediction of breast cancer molecular subtypes on DCE-MRI using convolutional neural network with transfer learning between two centers
**46**	67	17	2021	Breast cancer diagnosis/detection on MR	Radiology	Artificial Intelligence Applied to Breast MRI for Improved Diagnosis
**47**	67	13	2019	Review article	American Journal of Roentgenology	New Frontiers: An Update on Computer-Aided Diagnosis for Breast Imaging in the Age of Artificial Intelligence
**48**	67	17	2020	Radiomics, Breast cancer treatment	Ebiomedicine	MRI-based machine learning radiomics can predict HER2 expression level and pathologic response after neoadjuvant therapy in HER2 overexpressing breast cancer
**49**	65	9	2017	Breast cancer diagnosis/detection on US	Ultrasonography	Clinical application of S-Detect to breast masses on ultrasonography: a study evaluating the diagnostic performance and agreement with a dedicated breast radiologist
**50**	65	16	2020	Breast cancer diagnosis/detection on MG	Radiology Artificial Intelligence	Improving Breast Cancer Detection Accuracy of Mammography with the Concurrent Use of an Artificial Intelligence Tool
**51**	62	12	2019	Breast cancer diagnosis/detection on MG	Academic Radiology	Transfer Learning From Convolutional Neural Networks for Computer-Aided Diagnosis: A Comparison of Digital Breast Tomosynthesis and Full-Field Digital Mammography
**52**	62	6	2014	Breast cancer diagnosis/detection on MG	International journal of computer assisted radiology & surgery	Optimization of breast mass classification using sequential forward floating selection (SFFS) and a support vector machine (SVM) model
**53**	61	6	2013	Breast cancer diagnosis/detection on MR	Medical Physics	Automated fibroglandular tissue segmentation and volumetric density estimation in breast MRI using an atlas-aided fuzzy C-means method
**54**	61	10	2018	Review article	World Journal of Radiology	Artificial intelligence in breast ultrasound
**55**	61	12	2019	Radiomics, Breast cancer diagnosis/detection on MG	Radiology	Digital Mammography in Breast Cancer: Additive Value of Radiomics of Breast Parenchyma
**56**	59	30	2022	Radiomics, Breast cancer treatment	European Radiology	Deep learning radiomics of ultrasonography can predict response to neoadjuvant chemotherapy in breast cancer at an early stage of treatment: a prospective study
**57**	59	20	2021	Screening	European Radiology	Identifying normal mammograms in a large screening population using artificial intelligence
**58**	57	29	2022	Radiomics, Breast cancer diagnosis/detection on MR	European Radiology	Radiomic machine learning for predicting prognostic biomarkers and molecular subtypes of breast cancer using tumor heterogeneity and angiogenesis properties on MRI
**59**	56	12	2019	Breast cancer diagnosis/detection on MR	Academic Radiology	Automatic Breast and Fibroglandular Tissue Segmentation in Breast MRI Using Deep Learning by a Fully-Convolutional Residual Neural Network U-Net
**60**	53	3	2006	Breast cancer diagnosis/detection on MR	European Radiology	Cluster analysis of signal-intensity time course in dynamic breast MRI: does unsupervised vector quantization help to evaluate small mammographic lesions?

**61**	52	10	2019	Radiomics, Breast cancer diagnosis/detection on MG	Journal of the American College of Radiology	Added Value of Radiomics on Mammography for Breast Cancer Diagnosis: A Feasibility Study
**62**	51	9	2018	Breast cancer risk prediction	Journal of the American College of Radiology	Prediction of Occult Invasive Disease in Ductal Carcinoma in Situ Using Deep Learning Features
**63**	50	50	2023	Breast cancer diagnosis/detection on MG	Radiology	Automation Bias in Mammography: The Impact of Artificial Intelligence BI-RADS Suggestions on Reader Performance
**64**	49	4	2013	Breast cancer risk prediction	Academic Radiology	Prediction of Near-term Breast Cancer Risk Based on Bilateral Mammographic Feature Asymmetry
**65**	48	2	1999	Breast cancer risk prediction	Academic Radiology	Effect of patient history data on the prediction of breast cancer from mammographic findings with artificial neural networks
**66**	48	10	2019	Radiomics, molecular subtypes	European Radiology	Radiomic analysis of imaging heterogeneity in tumours and the surrounding parenchyma based on unsupervised decomposition of DCE-MRI for predicting molecular subtypes of breast cancer
**67**	48	24	2022	Radiomics, breast cancer treatment	European Journal of Nuclear Medicine and Molecular imaging	Co-clinical FDG-PET radiomic signature in predicting response to neoadjuvant chemotherapy in triple-negative breast cancer
**68**	47	4	2013	Screening	Journal of digital imaging	Automated Extraction of BI-RADS Final Assessment Categories from Radiology Reports with Natural Language Processing
**69**	47	7	2017	Screening	Journal of biomedical informatics	Automated annotation and classification of BI-RADS assessment from radiology reports
**70**	46	12	2020	Breast cancer diagnosis/detection on MR	Artificial intelligence in medicine	Multi-planar 3D breast segmentation in MRI via deep convolutional neural networks
**71**	46	12	2020	Review article	Breast	Machine learning with multiparametric magnetic resonance imaging of the breast for early prediction of response to neoadjuvant chemotherapy
**72**	46	15	2021	Radiomics, Breast cancer diagnosis/detection on US	European Radiology	Clinical value of radiomics and machine learning in breast ultrasound: a multicenter study for differential diagnosis of benign and malignant lesions
**73**	44	15	2021	Screening	European Radiology	Can artificial intelligence reduce the interval cancer rate in mammography screening?
**74**	44	11	2020	Screening	Journal of the American College of Radiology	Improving Workflow Efficiency for Mammography Using Machine Learning
**75**	44	15	2021	Molecular subtypes	European Radiology	Deep learning with convolutional neural network in the assessment of breast cancer molecular subtypes based on US images: a multicenter retrospective study
**76**	44	2	2004	Breast cancer diagnosis/detection on MR	European Radiology	Application of artificial neural networks to the analysis of dynamic MR imaging features of the breast
**77**	43	22	2022	Screening	Radiology	An Artificial Intelligence-based Mammography Screening Protocol for Breast Cancer: Outcome and Radiologist Workload
**78**	43	11	2020	Metastasis prediction	Academic Radiology	Preoperative Prediction of Ancillary Lymph Node Metastasis in Breast Carcinoma Using Radiomics Features Based on the Fat-Suppressed T2 Sequence
**79**	42	8	2019	Radiomics, breast cancer treatment	Journal of medical imaging & radiation sciences	Personalized Breast Cancer Treatments Using Artificial Intelligence in Radiomics and Pathomics

**80**	42	4	2012	Breast cancer diagnosis/detection on MR	Computer & Mathematics	Support vector machine for breast MR image classification
**81**	42	11	2020	Breast cancer treatment	International journal computer assisted radiology & surgery	Multi-input deep learning architecture for predicting breast tumor response to chemotherapy using quantitative MR images
**82**	40	8	2019	Breast cancer diagnosis/detection on MG	International journal computer assisted radiology & surgery	Classification of contrast-enhanced spectral mammography (CESM) images
**83**	39	13	2021	Survey	Journal of the American College of Radiology	Artificial Intelligence in Screening Mammography: A Population Survey of Women's Preferences
**84**	39	10	2020	Metastasis prediction	Clinical Breast Cancer	Convolutional Neural Network Detection of Axillary Lymph Node Metastasis Using Standard Clinical Breast MRI
**85**	39	20	2022	Screening	Radiology	Artificial Intelligence Evaluation of 122 969 Mammography Examinations from a Population-based Screening Program
**86**	38	10	2020	Metastasis prediction	Academic Radiology	Deep Learning Signature Based on Staging CT for Preoperative Prediction of Sentinel Lymph Node Metastasis in Breast Cancer
**87**	38	13	2021	Review article	European Radiology	AI-enhanced breast imaging: Where are we and where are we heading?
**88**	36	7	2019	Review article	American Journal of Roentgenology	Artificial Intelligence for Breast MRI in 2008-2018: A Systematic Mapping Review
**89**	36	12	2021	Radiomics, metastasis prediction	European Radiology	Preoperative prediction of axillary sentinel lymph node burden with multiparametric MRI-based radiomics nomogram in early-stage breast cancer
**90**	35	12	2021	Breast cancer diagnosis/detection on MG	European Radiology	Impact of artificial intelligence support on accuracy and reading time in breast tomosynthesis image interpretation: a multi-reader multi-case study
**91**	35	4	2016	Breast cancer diagnosis/detection on MG	Journal of biomedical informatics	Using automatically extracted information from mammography reports for decision-support
**92**	35	18	2022	Molecular subtypes	European Radiology	Predicting the molecular subtype of breast cancer and identifying interpretable imaging features using machine learning algorithms
**93**	35	18	2022	Radiomics, metastasis prediction	European Radiology	Radiomics model based on shear-wave elastography in the assessment of axillary lymph node status in early-stage breast cancer
**94**	35	18	2022	Radiomics, breast cancer treatment	European radiology	Intratumoral and peritumoral radiomics for preoperative prediction of neoadjuvant chemotherapy effect in breast cancer based on contrast-enhanced spectral mammography
**95**	34	17	2022	Screening	Radiology	Stand-Alone Use of Artificial Intelligence for Digit Mammography and Digital Breast Tomosynthesis Screening: A Retrospective Evaluation
**96**	34	9	2020	Breast cancer diagnosis/detection on MG	European Radiology	Digital breast tomosynthesis versus digital mammography: integration of image modalities enhances deep learning
**97**	34	11	2021	Radiomics, review article	Current oncology	Recent Radiomics Advancements in Breast Cancer: Lessons and Pitfalls for the Next Future
**98**	33	8	2020	Breast cancer diagnosis/detection on MG	Journal of digital imaging	A Multi-million Mammography Image Dataset and Population-Based Screening Cohort for the Training and Evaluation of Deep Neural Networks-the Cohort of Screen-Aged Women (CSAW)

**99**	32	8	2020	Breast density	Journal of the American College of Radiology	Multi-Institutional Assessment and Crowdsourcing Evaluation of Deep Learning for Automated Classification of Breast Density
**100**	30	10	2021	Breast cancer diagnosis/detection on MR	Radiology Artificial Intelligence	Improved Classification of Benign and Malignant Breast Lesions Using Deep Feature Maximum Intensity Projection MRI in Breast Cancer Diagnosis Using Dynamic Contrast-enhanced MRI
